# Supplementary material for: Novel DNA Markers for Identification of Actinobacillus pleuropneumoniae
Source: Microbiol Spectr. 2022 Jan 5;10(1):e01311-21. doi: 10.1128/spectrum.01311-21 (PMC8729771; doi:10.1128/spectrum.01311-21)

Supplementary data

Nucleotide sequence of novel *A. pleuropneumoniae*-specific marker candidates and representative gel electrophoresis results, showing the presence of selected markers in various strains of *A. pleuropneumoniae* and their specificity to only *A. pleuropneumoniae* are shown.

Nucleotide sequence of each marker candidate.

1. *apxIVA*-1

TTTGCTCATTCGATAAACGAATATTTTCTATTTTATGATCTTGGTGTGAATACCAA
TTTTGAACCGTGACTTTATCCTCACTTAATAATGATTTAATAATCAAATCGTTATT
TTCTCGGCTAAACCAAAGTTCGGATAAATTAATATCAGTAAATTTTAAGGTGTCG
ATATCTCTTGCGCGGTTATCATTATTGGTATCTTCATAAACGATATCCTGTCCGTG
TCCTTTGCTAAAGATATAGGTGTCCGCACCGTAGCCACCTCTTAGAATATCATTA
CCTTTGCCGCCGATGAGGGTGTCATCGCCATTACCGCCATTAACCGTATCATTAC
CCGCACCGGCATCAATCACCGAGTTACGTCCCCAGTCGTTGATATTATCATC

2. *apxIVA*-2

TCCAATTTGTCAAATTGATAATCTACATGGTTGTAGAAGGATTTTATCGTGACCG
AATCCGTATCATGATAACCGAATAACATTAAGTCATTATCTACTCGTCGGAATTT
CACTTCCGCATAATT

3. *apxIVA*-3

GCACCGTAGCTGCCTTTTAACGTATCATTACCCTCTCCGCCGATAAGTAGATCAT
CACCTGCACTACCCGATAAGTTATTATCTTTTTGGCTACCTAACAAAATATTATTT
TTTTCATCGCCTTTTAAATCCGTCGATTCTGCAAAAATTACTCCAGCCTCTCTTAA
ACGGGCAATAGTTAAGTTAGAAGTAGCCGCCCAGTTTTCATAAAATCCTTGTGCT
TTAGCATAATCTATATAACGGCTTAACAGTAATAGCCCTTCTTTCCATTCCGTAG
GATCGTTAAAGAAAGAAAGGTATTCGCTTAAATCATATAATGCATTTTC

4. *apxIVA*-4

CGTTTTAAGTAATGTTTTATCATAATGTTGATACTGTAAATCCGTTGCCGCCCATT
TATCTAAAATGGCAGGCAATAATTCTCGTTGTGCCTGAAAATCGGAGGCCTTAGT
GTACTGTTGTAATAAAGCAGCCAACTCCTCAGAAAGTGCGGCGGCTTCACGCAA
ATCCCGAACCCGACCGGTGCCGGTTAGATTAATTGTGCGACGTTGTTGCTCGGTT
AAATTTAACGCTTCGGTAAATCGGCTATAAAACGGGTTAAAACTGAAATTTAAAT
CGCCCATTTGTGCAAAAGTACCGTCCGTTTTTTCATACTT

5. *apxIVA*-5

CTCATTCGCTTCACTAAAACCGTTTTGATTTAAATCCCGCCAAATTTGGAGTTTAG
AAAACAGCTTATCATTTTGGTCGATACGCTGATCTTGGTTTGTATCCAAATCGGCT
AATG

6. *eamA*

ACACTTCAAGTCGGCACTGTCGGAATATGTTGCGGAACTTATTCTCTGCTATTTG
AAAATTTTCCTGAAACGATTTCTATAGAAGTGTGGTTATGGTTCACCGCCAGTTT
ACTCATTGCAACAAATCTTAGAATGCTTATACAAACTATCGGACAAAAATATTGT
CACGTCACTAACGCTGCAATTATTATGATTCTTGAGCC

7. *nusG*

CAAGTAGAAAAACAATCGTAATGGCTTTGTGATTTTATAAAATAAGATTGAAATT
AAGCGGTTTATTTTCAAAGAATATTTGCAAAAATAAGTTTAAAAATAACCGCTTT
TCTTATTCCACAAAGTGTTTTTTATCGGC

8. *sppA*

GTAAACCAACGACGTAAAGCGAATAACGAAGCGGAAATCGAACACTTTAGAAG
CCAATGGATGATTGATAGCGACGATAGTCTGTTCGGTTCATTGCTAAAAGGC

9. *xerD*

CCGACGTATTTTCAGATAATCCGTGTTCTTGCCATAACGTATCTAAAAACTGTTC
GATAATAGGATCTAAATTTTTCATGAAGCTACTTTTATTCCTAGATATTCTATTAT
TCTAACAGAATAACTTCACTTTAGAGAAAAGTACAAAT

10. *ybbN*

CACAAGTGCCGGTCGTGTTTAATTTTATCTCGCCGAGAGAAGTGGCTTCATTGGA
AATGGATTCATTATTACGCCGGTTGGCGGATGAAAATCCGGCACAATTTTTATTG
GCAACCGTGAATTGTGA

11. *ycfL*

AGCCATAAACTCAACCAAGGTTGCATCGATATATTGTTATTGGTTTGTGTAATGC
CTTGATTATCATACCAAAACAATTTATAGGATAAGTTAAGCGGATA

12. *ychJ*

ATAAGCGGTTATTTTTTCAAAATTCTTTGCAAAATTAACCGCTTGTATTTGAGGAT
TAGGCTAAATAGGCGCCACAGCAATGTTTGAATTTTTTACCGGAACCGCATACGC
AATTTTGTTTTTGCGAAGGGAGTGGTACG

Representative Gel Electrophoresis results

PCR of each marker was performed using primers listed in Table 4. Genomic DNA of the following *A. pleuropneumoniae* and other bacteria was used as template.

1. ATCC 27088^T,
2. ATCC 27089
3. ATCC 27090
4. ATCC 33377
5. *A. pleuropneumoniae* L20
6. ATCC 55454
7. ATCC 33590
8. *A. pleuropneumoniae* 405
9. ATCC 9346

10. ATCC 13372
11. ATCC 27072
12. ATCC 15557
13. ATCC 19417
14. ATCC 33391
15. ATCC 43765
16. No template control

PCR products were subjected to agarose gel electrophoresis and ethidium bromide staining. Representative gel pictures are shown below. Expected sizes of PCR product are shown in parentheses.

1. *eamA* (192 bp)

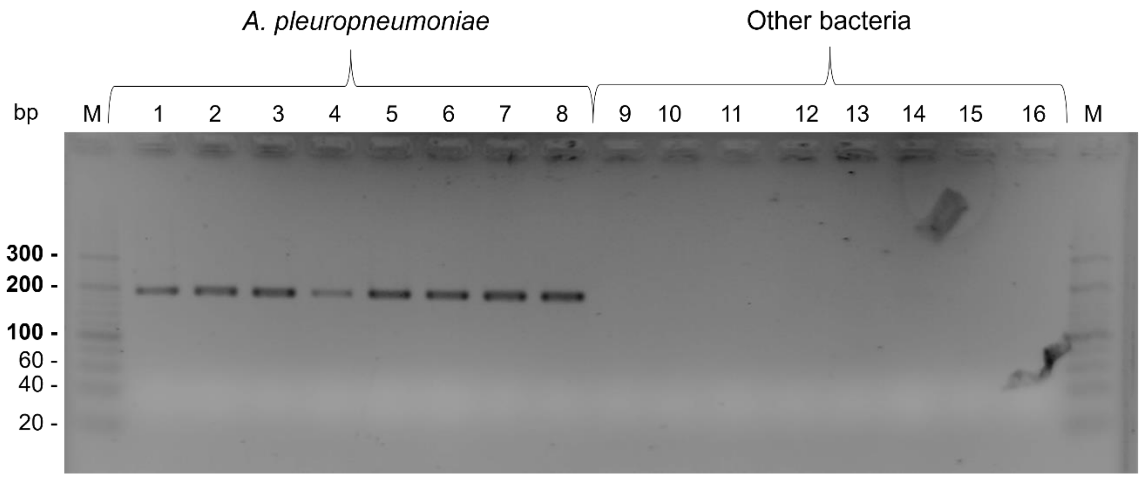

2. *nusG* (117 bp)

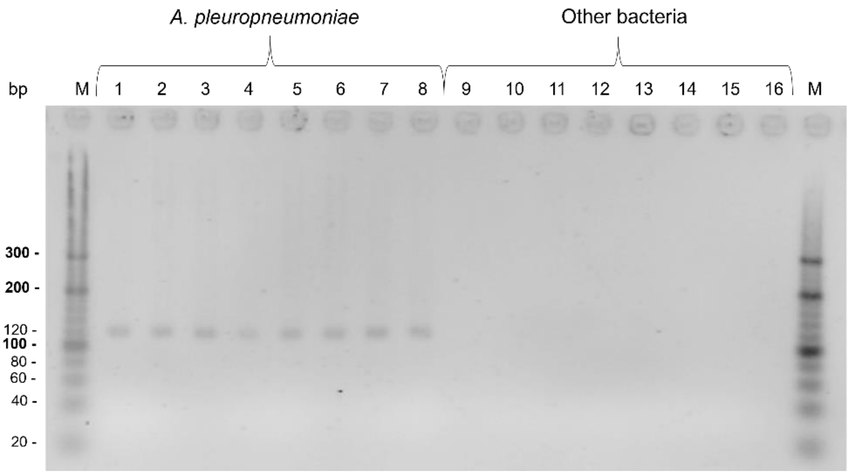

3. *sppA* (83 bp)

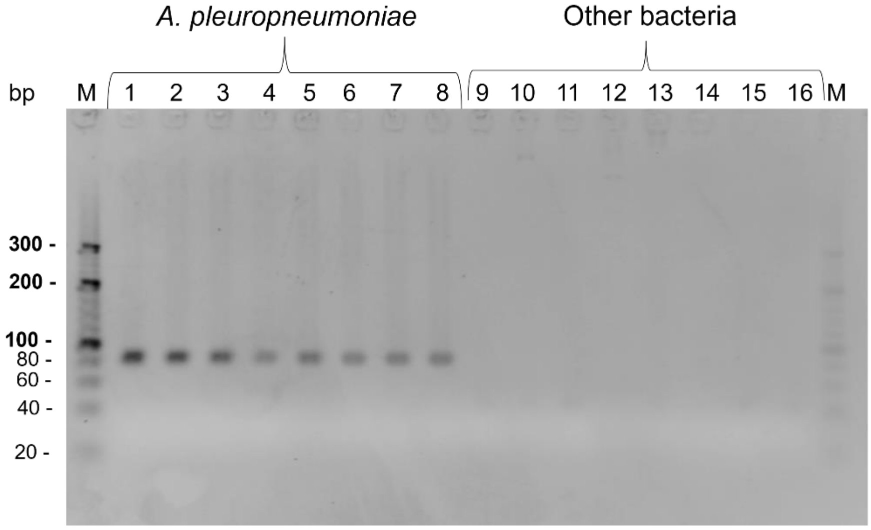

4. *xerD* (74 bp)

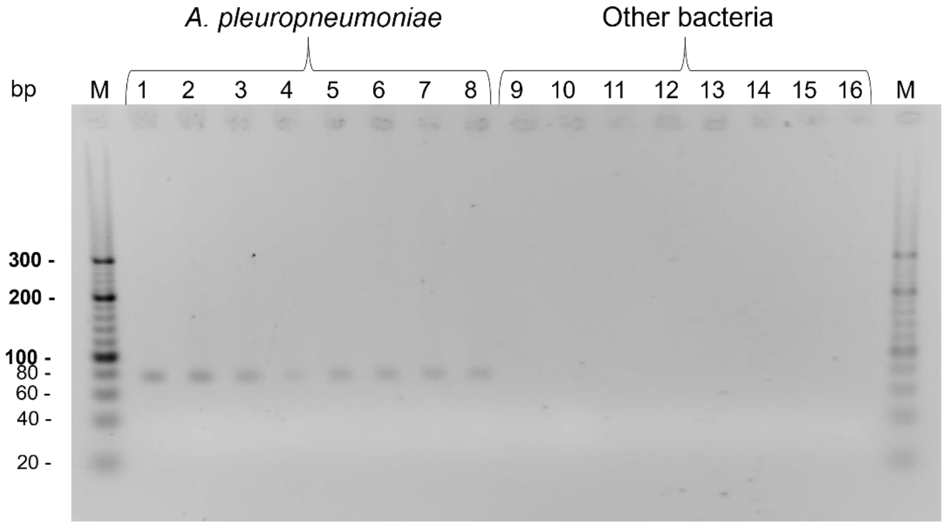

5. *ybbN* (58 bp)

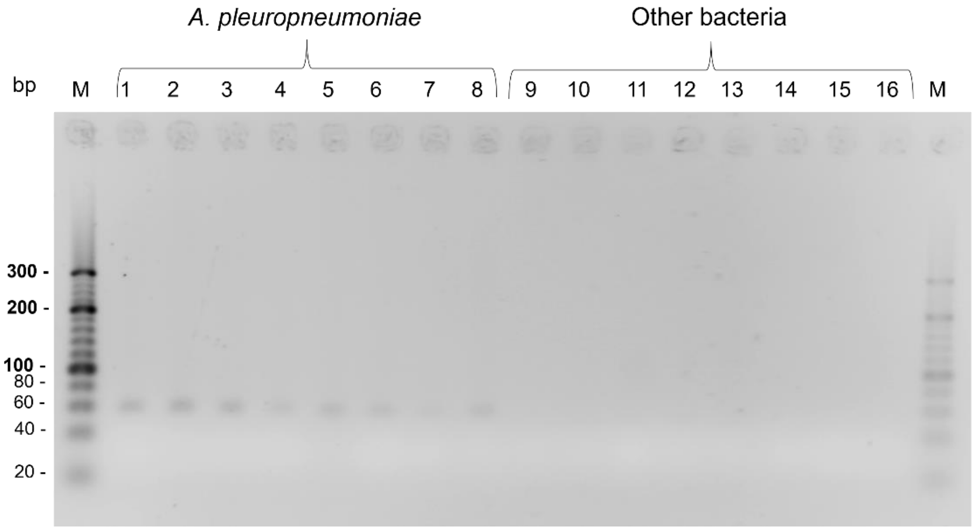

6. *ycfL* (54 bp)

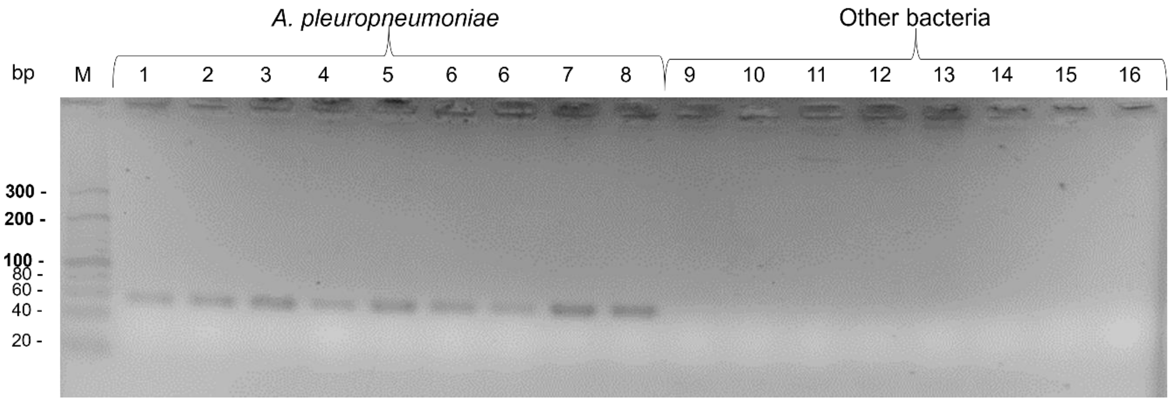

7. *ychJ* (66 bp)

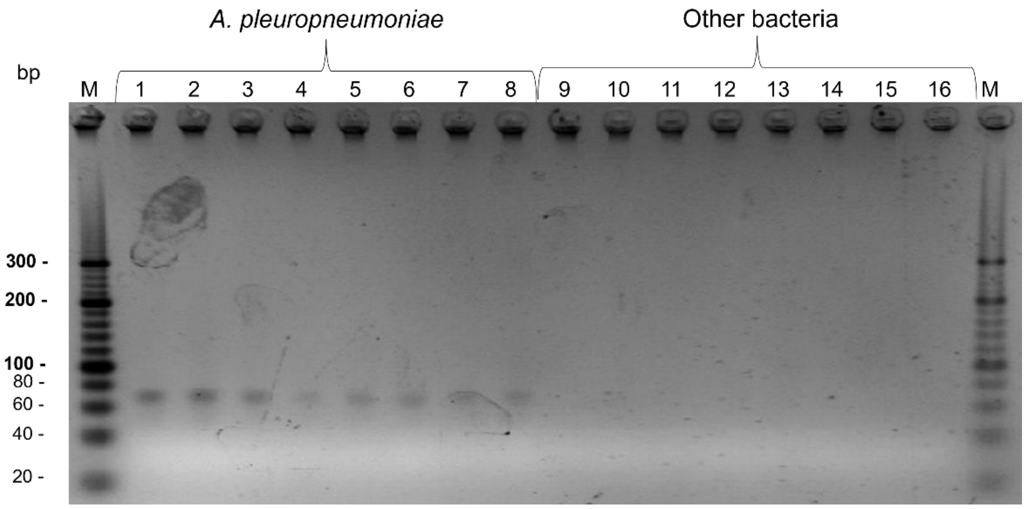

Supplement: SUPPLEMENTAL FILE 1 — Supplemental material. Download SPECTRUM01311-21_Supp_1_seq4.pdf, PDF file, 0.3 MB [file spectrum01311-21_supp_1_seq4.pdf]
